# Identification and Evaluation of Thermotolerance in Broccoli Seedlings Based on a Multi-Trait Phenotyping System

**DOI:** 10.3390/biology14081093

**Published:** 2025-08-21

**Authors:** Xuaner Li, Yongyu Zhao, Tiemin Xu, Xigang Feng, Fengqing Han, Dongna Wen, Yumei Liu, Wenzheng Gao, Zhiwei Zhao, Zhansheng Li

**Affiliations:** 1 State Key Laboratory of Vegetable Biobreeding, Institute of Vegetables and Flowers, Chinese Academy of Agricultural Sciences, Beijing 100081, Chinafengxigang@caas.cn (X.F.); wendongna0713@163.com (D.W.); liuyumei@caas.cn (Y.L.); gaowenzheng1996@163.com (W.G.); 2 Shouguang R&D Center of Vegetables, Chinese Academy of Agricultural Sciences, Shouguang 262700, China; zgys9814@126.com

**Keywords:** broccoli, heat tolerance, physiological indicator, electrical conductivity, seeding

## Abstract

To establish a systematic approach for evaluating heat tolerance at the seedling stage in broccoli, we investigated 14 representative cultivars cultivated in China. Physiological indicators such as electrical conductivity, malondialdehyde, proline, and chlorophyll content were measured before and after heat stress, alongside phenotypic scoring of heat injury, to characterize the differential thermotolerance among genotypes. The results indicated that significant differences (*p* < 0.05) in heat tolerance at the seedling stage were observed among different broccoli cultivars after heat stress treatment at 40 °C. Among them, Yanxiu exhibited the strongest heat tolerance, followed by Meiqing, Naihanyouxiu, Meiao 7172, Feicui 5, Guowang 11, Zheqing 80, Zhongqing 15, Zhongqing 318, Zhongqing 319, and Qianghan. Lvxiong 90, Zhongqing 11, and Zhongqing 16 were the least heat tolerant. Pearson’s correlation analysis demonstrated that the seedling heat tolerance of different broccoli cultivars was significantly negatively correlated with electrical conductivity, with a correlation coefficient of 0.542 (*p* < 0.05). In this study, a rapid and robust method for determining the heat resistance response of broccoli was established, providing a scientific basis and technical support for the identification of the heat resistance of new broccoli cultivars and the selection and breeding of heat-tolerant cultivars in China.

## Simple Summary

As extreme heat events become more common due to global climate change, the ability of crops to survive high temperatures is increasingly important. Broccoli, a cool-season vegetable, is especially vulnerable to heat during the seedling stage, which can affect yield and quality. In this study, we developed a quick and effective way to identify which broccoli cultivars are more heat tolerant. By examining plant health indicators like cell damage and chlorophyll levels, we tested 14 common broccoli varieties and found that some, like ‘Yanxiu’, recovered well from heat, while others were more sensitive. Our findings can help farmers and breeders choose better varieties for hot climates and guide future development of heat-tolerant broccoli.

## 1. Introduction

Broccoli (*Brassica oleracea* L.var. *italic*), one of *Brassica oleracea* family of vegetables, has been present in China since the 1980s. This vegetable is renowned for its rich nutritional content, including high levels of vitamins, proteins, and various other nutrients. Consequently, the cultivation area for cauliflower and broccoli has seen rapid expansion in China over the past three decades. In 2021, according to data from the Food and Agriculture Organization (FAO) of the United Nations, the planting area for cauliflower and broccoli in China reached 484,000 hectares, with a total output of approximately 9.606 million tons [1,2]. However, global warming has led to an increase in the frequency and intensity of extremely hot weather. This rise in temperature poses the risk of high-temperature stress in the agricultural sector, which could potentially threaten the sustainable development of the industry [3]. High-temperature stress, common among cool-season vegetables like broccoli, can lead to a variety of detrimental effects. These include abnormal flower bud differentiation, the formation of hairy balls, morphological deformities, and issues with pod leaves. These conditions can result in bud wilting and other adverse traits, causing significant economic losses or even complete crop failure [4]. In northern China, during the months of June and July, when autumn broccoli is typically sown, the seedling stage coincides with the summer and autumn seasons, which are characterized by high temperatures. This timing makes broccoli seedlings particularly vulnerable to high-temperature damage, increasing the risks associated with planting. Current research on plant heat tolerance predominantly concentrates on C3 plants such as maize, rice, and wheat, with *Brassica napus* being the primary cruciferous crop studied [5,6,7,8,9,10,11]. Adequate watering can enhance photosynthesis and promote early vegetative growth in broccoli. However, high temperatures above 38 °C disrupt chlorophyll biosynthesis and trigger photosynthetic pigment degradation in broccoli seedlings. Studies have shown that both chlorophyll a and chlorophyll b contents decrease significantly under heat stress, with chlorophyll b being more sensitive—leading to reductions in total chlorophyll content of up to 12% under high-temperature regimes [12]. Heat exposure further elevates oxidative stress markers such as malondialdehyde and electrolyte leakage, reflecting damage to cellular membranes and diminished physiological integrity [13]. Young broccoli plants exhibit stage-dependent metabolic responses; for instance, seedlings show dramatic increases in proline accumulation (up to 587%) and other antioxidative compounds under heat exposure [14]. The combined effects of pigment loss, oxidative stress, and metabolic imbalance contribute to decreased photosynthetic efficiency, impaired growth, and potential yield penalties under prolonged heat episodes. These findings underscore the susceptibility of broccoli—particularly at the seedling stage—to thermal stress and highlight the importance of evaluating chlorophyll stability, membrane integrity, and stress metabolites when screening for heat resilience.

Recent research indicates that multiple factors, including genotypes, environmental conditions, and the interactions between these two, all play a role in determining a plant’s tolerance to high temperatures. These factors can lead to substantial changes in the plant’s morphology, yield, and reproductive capabilities [15]. Currently, most methods for identifying heat tolerance in Brassica vegetables focus on the later stages of growth, such as the late stages of vegetative growth or during reproductive development [9,16]. These methods are often characterized by their lack of stability, lengthy timeframes, and high levels of difficulty in detection. There is a significant gap in the availability of efficient and systematic methods for rapidly identifying heat tolerance, which is crucial for both scientific research and vegetable breeding efforts. Therefore, there is an urgent need to develop a technology system that is efficient, rapid, and reliable for detecting heat tolerance in *Brassica oleracea*, which includes broccoli and other related vegetables. Such a system would be invaluable for advancing breeding and improvement efforts in this important crop group.

In this study, different domestic broccoli cultivars planted in China were investigated and performance was evaluated before and after exposure to stress, such as heat-tolerance grade, electrical conductivity, and contents of malondialdehyde, proline, and chlorophyll. By analyzing the correlations between morphological traits and physiological indices, the study successfully developed a systematic, rapid, and reliable method for assessing broccoli’s heat resistance. This approach offers a scientific foundation and technical support for identifying and selecting heat-tolerant broccoli cultivars in China, aiding in the development of improved varieties better suited to changing climate conditions.

## 2. Materials and Methods

### 2.1. Materials

A total of 14 broccoli cultivars that have been cultivated in China over the past decade were collected and subjected to testing. Fourteen broccoli varieties were obtained from the Institute of Vegetables of the Chinese Academy of Agricultural Sciences, commercial seed companies such as SAKATA (Yokohama, Kanagawa, Japan), MITSUO (Tokyo, Japan), and China’s national breeding projects (Table 1). These genotypes represent typical varieties with varying maturity levels in China, providing a diverse sample for the study. Each cultivar was represented by a total of 30 seedlings, which were subjected to both phenotypic and physiological evaluations. The experimental design included five biological replicates per treatment for each cultivar. For each biological replicate, three technical measurements were performed to assess the physiological parameters.

### 2.2. Methods

#### 2.2.1. Pretreatment

The 14 broccoli cultivars, labeled B1 to B14, were sown on 29 December 2022. Seedlings were grown in a greenhouse under ambient temperature (22–25 °C, 16 h light/8 h dark) until the 5–6 leaf stage, then transferred to a programmable artificial climate incubator (RXZ-300B, Ningbo Southeast Instrument, Ningbo, China). After the seedlings had grown to the stage with 5 to 6 leaves, they were moved to an incubator. The seedlings were transferred to a programmable artificial climate incubator, which allows precise regulation of temperature, light, and humidity. The chamber was set to a 40 °C light/36 °C dark cycle with a 16 h light/8 h dark photoperiod and was equipped with a ventilation system to ensure uniform environmental conditions across all samples. After 72 h of treatment under these conditions, the phenotypic characteristics and physiological indices of the seedlings were evaluated and documented to determine the effects of heat stress on each cultivar (Table 2).

#### 2.2.2. Phenotypic Investigation

**Figure 1:**
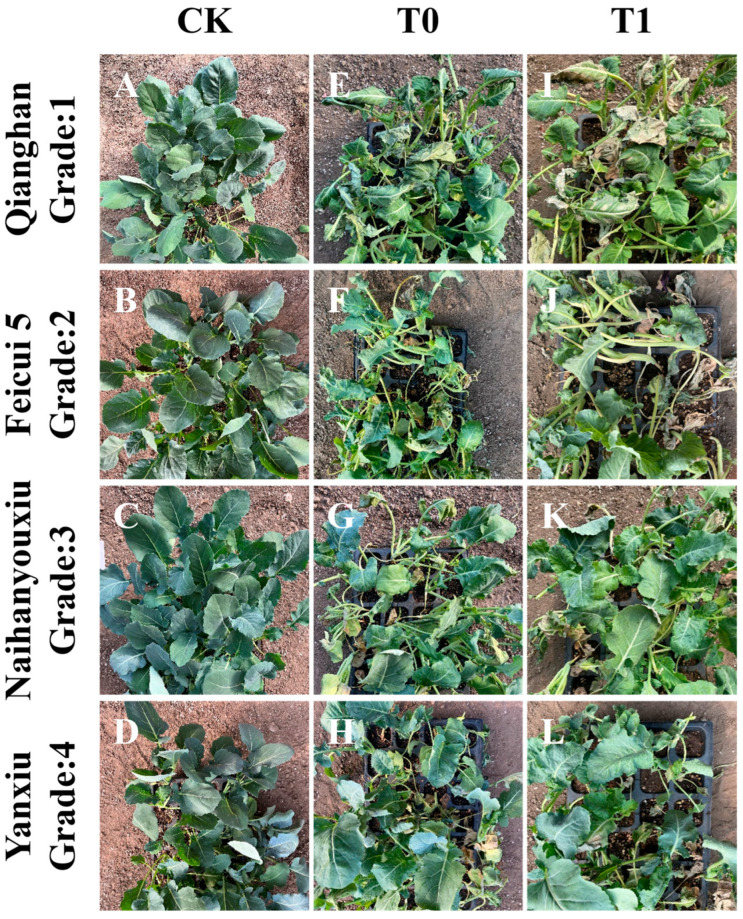
Phenotypic performance of four representative broccoli cultivars under heat stress treatment. (**A**–**D**) Control group (CK): morphology before heat stress. (**E**–**H**) Immediately after 72 h of high-temperature treatment (T0). (**I**–**L**) Three days after recovery at ambient temperature (T1). From top to bottom: ‘Qianghan’ (Grade 1), ‘Feicui 5’ (Grade 2), ‘Naihanyouxiu’ (Grade 3), and ‘Yanxiu’ (Grade 4).

After experiencing high-temperature stress, the broccoli seedlings were given a 3-day recovery period at normal temperature, room temperature (22–25 °C), photoperiod (16 h of light/8 h of darkness), and all samples were under the same environmental conditions, to observe their ability to recover from the stress. After the heat stress treatment and a 3-day recovery period under ambient conditions, the broccoli seedlings were assessed for visible damage symptoms. A trait-based phenotypic scoring system was employed, which evaluated seven specific traits associated with heat damage: wilting, drying, regreening, collapse, green loss, heart leaf change, and apical damage. Each trait was scored on a scale of 0 to 5, where 0 indicates severe damage and 5 represents no visible symptoms. The final score for each cultivar was calculated as the arithmetic mean of the seven trait scores. The definitions of individual scores were standardized using visual references (Figure 1) to ensure consistency. For instance, a score of 3 typically indicated moderate wilting or partial leaf yellowing, while a score of 5 indicated healthy, undamaged tissue. Representative phenotypic grades are shown in Figure 1 to visually support the scoring system. Grade 1 (Qianghan, panel I) represents severe wilting and dehydration, with heart leaves collapsed or necrotic and extensive chlorosis; Grade 2 (Feicui 5, panel J) shows moderate dehydration and collapse of leaf margins, with visible yellowing and shriveling; Grade 3 (Naihanyouxiu, panel K) indicates mild stress symptoms such as slight wilting and leaf margin curling, while heart leaves remain intact; and Grade 4 (Yanxiu, panel L) reflects minimal visual damage, with only subtle edge discoloration or temporary wilting, and overall morphology well preserved.

#### 2.2.3. Measurement of Electrical Conductivity

The leaf samples from identical sections of 14 different broccoli varieties were extracted using a 1.0 cm diameter circular punch. Each 0.2 g of the circular leaf sections were then transferred into a test tube, followed by the addition of 10 milliliters of distilled water. To minimize mechanical leakage leaf disks were immediately immersed in distilled water, and preliminary trials showed that baseline conductivity remained stable across control replicates. The same procedure was used for all samples to ensure comparability. After the high-temperature stress treatment, leaf samples were collected and transferred into test tubes containing 10 mL distilled water. To ensure consistency in measurement conditions, the samples were incubated at 25 °C for 12 h before measuring the initial electrical conductivity (E_1_). Subsequently, the tubes were subjected to a 35 min boiling water bath. Once cooled, the electrical conductivity was remeasured and documented as E_2_. Additionally, the baseline electrical conductivity of the distilled water was also measured and recorded as E_0_. The relative electrical conductivity was computed using the formula [20,21]:

E = (E_1_ − E_0_)/(E_2_ − E_0_) × 100%

#### 2.2.4. Measurement of Malondialdehyde Content

The content of malondialdehyde was quantified using a specialized assay kit from Solarbio (Cat: #BC6410) Ltd. (Beijing, China). A leaf sample weighing 0.1 g was combined with 1.0 mL of extraction solution. The mixture was then finely ground on ice and subjected to centrifugation. Following centrifugation, the supernatant was carefully decanted, and the malondialdehyde detection reagent was added to it. This solution was then incubated in a water bath at 100 °C for a duration of 60 min before being centrifuged once more. After centrifugation, the supernatant was collected and its absorbance was measured at 532 nm and 600 nm, respectively. Utilizing these absorbance values, the malondialdehyde content for each sample was computed [22].

#### 2.2.5. Measurement of Proline Content

To determine the proline content, a 0.1 g leaf sample was processed using an assay kit supplied by Solarbio (Cat#BC0025) Co., Ltd. (Beijing, China). The sample was first mixed with 1 mL of extraction solution, ground on ice, and then the homogenate was incubated in a boiling water bath for 10 min. Subsequently, the mixture was centrifuged to separate the supernatant. After allowing the supernatant to cool, its absorbance was measured at 520 nm. This absorbance value was then used to interpolate the proline concentration from a preconstructed standard curve, thereby quantifying the proline content in each sample [23].

#### 2.2.6. Measurement of Chlorophyll Content

The extraction of chlorophyll followed previously validated methodologies. For this process, leaves from equivalent sections of broccoli seedlings were carefully selected. Fresh weight samples, each weighing 0.1 g, were finely ground using a mortar and pestle. The sample was first mixed with 1 mL of extraction solution, ground on ice, and then the homogenate was incubated in a boiling water bath for 10 min. To prevent photodegradation, the tubes were kept in the dark for 3 h. Following this incubation period the absorbance of the extracted solution was measured at 663 nm and 645 nm wavelengths, and the values were recorded as A663 and A645, respectively [24].

The total chlorophyll concentration was calculated using the following formula:

C_T_ (mg/g) = 8.02 × A_663_ + 20.21 × A_645_

#### 2.2.7. Statistical Analysis

Data analysis was conducted using SPSS version 18.0, and the results were expressed as mean ± standard deviation (SD) (*n* = 3). Statistical evaluation of the treatments was performed using paired t-tests. When normality was not met, Wilcoxon signed-rank tests were applied. To evaluate the magnitude of stress-induced changes, effect size analyses were conducted using Cohen’s d between control and heat-treated groups for each physiological parameter (electrical conductivity, malondialdehyde, proline, and chlorophyll) across all 14 cultivars. Bootstrapped 95% confidence intervals for each effect size were computed using the ‘effsize’ and ‘bootES’ packages in R version 4.3.2. Forest plots illustrating the effect sizes and their statistical significance were generated using the matplotlib and seaborn libraries in Python 3.11. A Cohen’s d value with a 95% confidence interval excluding zero was deemed statistically significant. Additionally, Pearson’s correlation analysis was applied to analyze the relationships between the responses of broccoli cultivars to heat stress and their physiological changes.

## 3. Results

### 3.1. Performance of Different Broccoli Cultivars After Treatment

To comprehensively assess heat tolerance, we evaluated each broccoli cultivar based on seven visual traits: green color loss, heart leaf change, leaf collapse, wilting, drying, apical damage, and regreening ability. The average of these individual trait scores was calculated to derive a final damage score for each cultivar. Among the 14 cultivars, ‘Zhongqing 16’ exhibited the highest average score (4.86), indicating minimal damage and superior recovery after heat stress. This was followed by ‘Zhongqing 11’ (4.71), ‘Lvxiong 90’ (4.14), and ‘Yanxiu’ (4.14), all of which maintained relatively intact leaf structures, limited apical damage, and strong regreening. In contrast, ‘Qianghan’ showed the lowest final score (2.57), characterized by extensive wilting, dehydration, and poor regreening capacity, suggesting high susceptibility to thermal stress. Other cultivars with low heat tolerance included ‘Zhongqing 16’, ‘Meiqing’, and ‘Zheqing 80’, each scoring ≤ 3.0. Notably, some cultivars demonstrated specific weaknesses in particular traits despite moderate overall scores. For example, ‘Guowang 11’ had a high degree of leaf collapse and drying, while ‘Feicui 5’ exhibited severe apical damage. These variations highlight the importance of multi-trait assessment in accurately distinguishing thermotolerance responses. Overall, the trait-based scoring approach enabled a detailed comparison of heat damage patterns and allowed classification of cultivars into tolerant, intermediate, and sensitive groups, thereby supporting more informed selection for heat-resilient breeding (Table 3).

### 3.2. Changes in Electrical Conductivity After Treatment of Different Broccoli Cultivars

**Figure 2:**
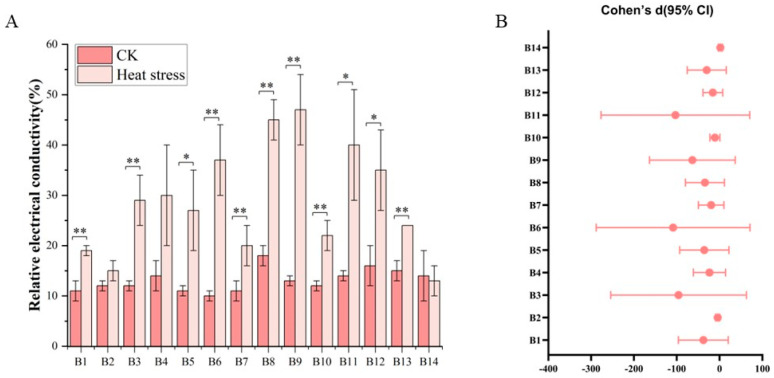
Electrical conductivity changes in 14 Broccoli cultivars under heat stress. (**A**) Relative electrical conductivity (%) of each cultivar before and after treatment. (**B**) Effect sizes (Cohen’s d) with 95% confidence intervals comparing CK and heat stress conditions for each cultivar. Significant differences were determined using paired *t*-tests (* *p* < 0.05; ** *p* < 0.01).

Figure 2 clearly depicts the significant differences in electrical conductivity among various broccoli seedling varieties before and after exposure to high-temperature stress. Before the high-temperature treatment, the electrical conductivity among the cultivars showed only slight variation, ranging from 8.23% to 19.42%, with an average of 13.02%. Cultivar B8, identified as ‘Zhongqing 11’, exhibited the highest initial conductivity, whereas cultivar B6, ‘Guowang 11’, displayed the lowest. Following the high-temperature treatment, an increase in electrical conductivity was observed across most cultivars, with the exception of B2, B4, and B14. The post-treatment conductivity values spanned from 9.68% to 54.17%, with an average of 28.71%. Cultivar B9, ‘Zhongqing 15’, experienced the greatest increase in conductivity, while B14, ‘Meiao 7172’, showed the least increase. Statistically significant differences in electrical conductivity were noted before and after the treatment for all cultivars, except for B2, B4, and B14 which did not exhibit significant changes.

In Figure 2B, this set of data shows a very consistent trend. The effect sizes of most of the electrical conductivity contents are negative and the confidence intervals are completely below 0, indicating that heat stress significantly reduces the values of these indicators. Specifically, the Cohen’s d values of the 13 electrical conductivity contents, B1, B2, B3, B4, B5, B6, B7, B8, B9, B10, B11, B12, and B13, are all negative and the corresponding 95% confidence intervals are all below 0, indicating that under heat stress conditions the electrical conductivity contents have significantly decreased compared to the control group CK. For example, the effect size of B8 is as high as −8.21, B6 is −5.41, and B3 is −5.10. These extremely large negative effect sizes show that heat stress has a strong inhibitory effect on multiple dimensions of electrical conductivity, and the statistical significance is very high. In addition, the confidence intervals of some electrical conductivity contents are very wide, such as B3, B6, and B11, with the upper and lower bounds spanning over 250, indicating that there is a large variation in these electrical conductivity contents among samples and may be affected by extreme values. However, the overall effect direction of these electrical conductivity contents is still clearly negative.

### 3.3. Changes in Malondialdehyde Content After Treatment of Different Broccoli Cultivars

**Figure 3:**
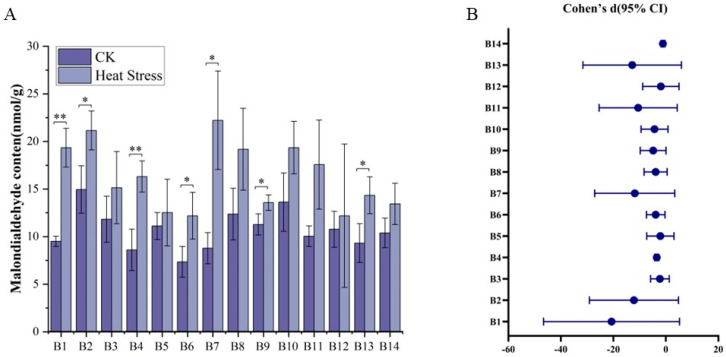
Changes in malondialdehyde content in 14 broccoli cultivars before and after heat stress. (**A**) Malondialdehyde levels (nmol/g) before and after treatment. (**B**) Effect sizes (Cohen’s d) with 95% confidence intervals between CK and heat stress conditions. Significant differences were determined using paired *t*-tests (* *p* < 0.05; ** *p* < 0.01).

It demonstrated that 14 broccoli cultivar seedlings displayed considerable alterations of malondialdehyde levels in response to high-temperature stresses (Figure 3). Before the application of high-temperature stress, the malondialdehyde content in the leaves of the broccoli cultivars exhibited slight variability, spanning from 5.73 nmol/g to 19.02 nmol/g, with an average concentration of 10.71 nmol/g. Cultivar B2, ‘Yanxiu’, had the highest malondialdehyde content, while B6, ‘Guowang 11’, had the lowest. After the high-temperature treatment, the malondialdehyde content in the leaves of cultivars B1 through B13 increased, with levels ranging from 6.81 nmol/g to 27.78 nmol/g and an average of 16.32 nmol/g. Notably, B9, ‘Zhongqing 15’, showed the most significant increase in malondialdehyde content, while B14, ‘Meiao 7172’, had the least significant increase. The comparisons of malondialdehyde levels before and after high-temperature treatment for all 14 cultivars revealed significant or highly significant differences for most cultivars, with the exception of B3, B5, B8, B10, B11, B12, and B14. Moreover, there were pronounced differences in malondialdehyde content among all varieties following the high-temperature treatment, indicating varied responses to stress within the cultivars tested.

As can be seen from Figure 3B, the Cohen’s d effect sizes are generally negative, indicating that the average level of malondialdehyde decreases under heat stress conditions, and the values in the CK are generally higher than those in the heat stress groups. Among them, B1 shows the strongest effect, with a Cohen’s d of −6.62, and a confidence interval completely below 0 indicating a highly significant difference between the two treatments. Similarly, B2, B4, B6, B7, B8, B9, B10, B11, and B13 also have significant negative effects, and the confidence intervals of these malondialdehyde content changes do not cross zero, indicating that the differences between CK and heat stress are statistically significant. This is especially evident in B4 and B14, with the lower limit of the confidence interval being negative infinity, highlighting the extremely strong differences in these malondialdehyde contents. Although the confidence intervals of some malondialdehyde contents include 0, their effect sizes still show a certain trend. For example, the Cohen’s d of B3 is −1.04, although the upper limit is slightly above 0, suggesting a certain directionality but not being significant. The same is true for B5 and B12, with Cohen’s d values of −0.53 and −0.26, respectively, indicating a relatively large uncertainty.

### 3.4. Changes in Proline Content of Different Broccoli Cultivars After Treatment

**Figure 4:**
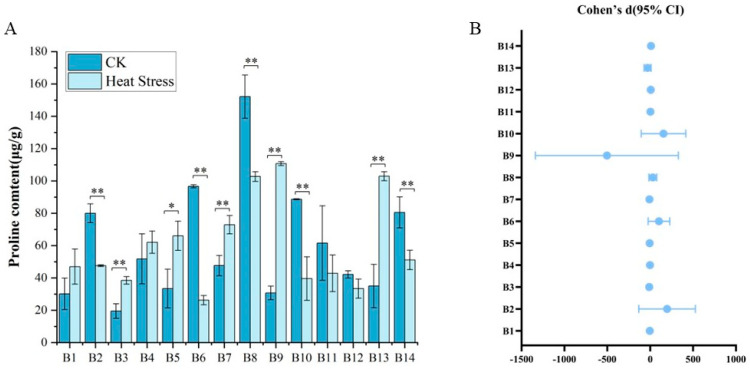
Proline content in 14 broccoli cultivars under heat stress. (**A**) Proline levels (μg/g) before and after treatment. (**B**) Effect sizes (Cohen’s d) and 95% confidence intervals for each cultivar comparing CK and heat stress. Significant differences were determined using paired *t*-tests (* *p* < 0.05; ** *p* < 0.01).

From Figure 4, we found that there were seven broccoli cultivar seedlings that showed significant fluctuations of proline contents in response to high-temperature treatments. Before the onset of high-temperature stress, the proline content in the leaves of the 14 broccoli cultivars displayed considerable diversity, with values ranging from 15.22 μg/g to 163.33 μg/g and an average of 60.7 μg/g. Cultivar B8, ‘Zhongqing 11’, had the highest initial proline content, while B3, ‘Qianghan’, had the lowest. After exposure to high temperatures, the proline content in the leaves of cultivars B1 through B14 either rose or fell, with measurements varying from 23.36 μg/g to 112.13 μg/g and an average of 60.26 μg/g. Notably, B9, ‘Zhongqing 15’, displayed the greatest increase in proline content, while B6, ‘Guowang 11’, had the lowest levels post-treatment. The comparisons of proline contents before and after high-temperature treatments for all 14 cultivars revealed significant or highly significant differences for most, except for B1, B4, B11, and B12. Furthermore, there were marked differences in proline content among all varieties following the high-temperature treatment, indicating a diverse response to thermal stress within the group of cultivars examined.

As shown in Figure 4B, among the varieties with an upward trend in proline content, B2, B6, B8, B10, B12, and B14 are the most representative. Notably, B6 has a Cohen’s d value as high as 33.20, with its confidence interval far from zero and the upper limit even exceeding 247, indicating an extremely significant positive effect. This effect suggests that under heat stress conditions, the proline level significantly increases, possibly reflecting the activation of osmotic adjustment mechanisms by plants to adapt to high-temperature environments. The effect sizes of B2, B8, B10, and B14 are all greater than three and their 95% confidence intervals are completely above zero, indicating that the changes in proline content of these varieties are highly statistically significant. Besides the significantly increasing varieties, B11 and B12 also show an upward trend, but their confidence intervals include zero or are close to zero. For instance, the upper limit of B11 is 7.5, but the lower limit is slightly below zero, suggesting that although the effect direction is positive the statistical significance is relatively low. Although the confidence interval of B12 is relatively wide, it is overall above zero and can be regarded as a relatively significant positive response. Conversely, the proline content of some varieties significantly decreases under heat stress. The Cohen’s d values of B3, B5, B7, B9, and B13 are all negative and their confidence intervals are completely below zero, indicating a clear downward trend under stress conditions possibly representing an inhibitory response of another type of proline metabolic or regulatory pathway. Among them, the effect size of B9 is −25.96, with the lower limit of the confidence interval as low as −1467, suggesting the possible presence of extreme values, but the downward trend is extremely significant. The Cohen’s d values of B3 and B5 are −5.33 and −3.07, respectively, also representing strong negative effects. Overall, the changes in proline content exhibit characteristics of “extreme” and bidirectionality, reflecting the complex mechanism of this important osmotic adjustment substance in stress responses.

### 3.5. Changes in Chlorophyll Content After Treatment of Different Broccoli Cultivars

**Figure 5:**
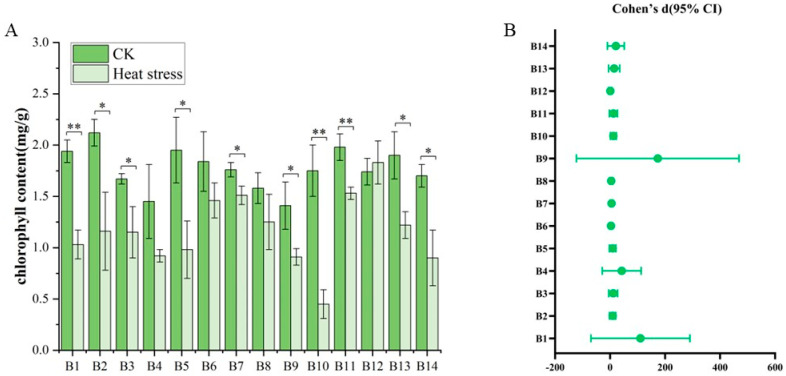
Chlorophyll content changes in 14 broccoli cultivars before and after heat stress. (**A**) Total chlorophyll content (mg/g) in CK and heat stress groups. (**B**) Effect sizes (Cohen’s d) with bootstrapped 95% confidence intervals. Significant differences were determined using paired *t*-tests (* *p* < 0.05; ** *p* < 0.01).

The results illustrated the variability in chlorophyll content among seedlings of 14 broccoli cultivars before and after exposure to high-temperature stresses (Figure 5). Before the high-temperature treatment, the chlorophyll content in the leaves of the cultivars showed relatively slight differences, spanning from 1.03 mg/g to 2.25 mg/g, with an average of 1.77 mg/g. Cultivar B5, ‘Lvxiong 90’, had the highest chlorophyll content, whereas B4, ‘Feicui 5’, had the lowest. After the high-temperature treatment, the chlorophyll content in the leaves of the B1–B14 broccoli cultivars decreased across the board, with values ranging from 0.30 mg/g to 2.07 mg/g and an average of 1.17 mg/g. Post-treatment, B12, ‘Zhongqing 319’, had the highest chlorophyll content, while B10, ‘Zhongqing 16’, had the lowest. The comparison of chlorophyll levels before and after the high-temperature treatment for the B1–B14 cultivars revealed significant or highly significant differences for most, except for B4, B6, B8, and B12. Additionally, there were pronounced differences in chlorophyll content among all varieties following the high-temperature treatment, indicating a diverse response to thermal stress within the group of cultivars examined.

The results from Figure 5B show that most chlorophyll contents exhibit significant differences between the two groups, with generally large effect sizes. Overall, the effect sizes of chlorophyll contents such as B1, B2, B3, B5, B7, B10, B11, B13, and B14 all exceed two, with some even surpassing five, indicating that heat stress has a considerable impact on these chlorophyll contents. For instance, the effect size of B1 exceeds seven, and its lower confidence limit is also far above zero, suggesting that the difference between the two treatments is not only significant but also very strong. Similarly, the Cohen’s d values of B10 and B11 are 6.3 and 4.3, respectively, both showing extremely strong statistical effects. Such large effect sizes imply that heat stress may significantly disrupt or activate the physiological mechanisms related to these indicators. Secondly, there are some chlorophyll contents, such as for B6 and B8, where the effect sizes do not reach extreme values but their confidence intervals are entirely within the positive range, still indicating a moderate to strong statistical difference. These chlorophyll contents may be moderately disturbed by heat stress, and their change trends have a certain degree of consistency and reliability. It is worth noting that although the size of B9 effect is high, its confidence interval is very wide, indicating a large variation in this chlorophyll content among samples. This suggests that caution should be exercised when interpreting this chlorophyll content as there may be extreme values or large data fluctuations. The confidence interval of B4 is also relatively wide, falling into a similar situation. The only chlorophyll content that did not show a significant difference is B12. Its Cohen’s d is −0.52, and the 95% confidence interval extends from negative to positive values (−3.57 to 4.63), clearly including 0, indicating that the mean difference in chlorophyll content between the two treatment groups is not statistically significant. This may suggest that B12 is not sensitive to heat stress, or that the direction and magnitude of its response fluctuates greatly among different samples, thus resulting in an overall effect that is not significant.

### 3.6. The Correlation Analysis Among Different Indicators

**Figure 6:**
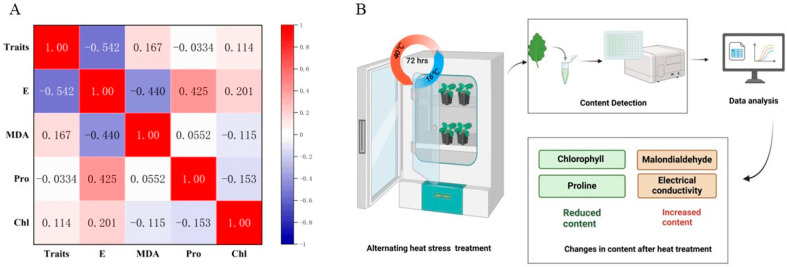
Correlation analysis and experimental workflow of physiological responses in broccoli cultivars under heat stress. (**A**) Pearson correlation matrix among heat tolerance traits and physiological indicators in 14 broccoli cultivars after high-temperature treatment. “Traits” represents the phenotypic score based on visible heat damage. Significant negative correlation was observed between electrical conductivity (E) and phenotypic traits (*r* = −0.542, *p* < 0.05). The color scale indicates correlation strength from −1 (blue) to +1 (red). MDA stands for malondialdehyde, Pro for proline, and Chl for chlorophyll. (**B**) Schematic illustration of the experimental workflow. Broccoli seedlings were exposed to alternating high temperatures (40 °C day/36 °C night) for 72 h in a growth chamber. Leaf samples were collected and analyzed for four physiological indicators: electrical conductivity and malondialdehyde [13], which increased under stress; and chlorophyll and proline, which generally decreased. The results were used for integrative phenotypic and physiological evaluation.

Broccoli seedlings subjected to alternating heat stress (40 °C day/36 °C night, 72 h; Figure 6B) exhibited clear physiological and biochemical changes, as evidenced by content detection and data analysis. Among the four measured indicators—electrical conductivity (electrical), malondialdehyde [13], proline, and chlorophyll—electrical and malondialdehyde increased significantly in most cultivars, while chlorophyll and proline tended to decrease after treatment, though the latter showed cultivar specific variability. As illustrated in Figure 6A, Pearson correlation analysis revealed a significant negative correlation between electrical and phenotypic heat tolerance scores (r = −0.542, *p* < 0.05), supporting its role as a reliable indicator of membrane damage under thermal stress. Malondialdehyde content was moderately correlated with electrical (r = −0.440), indicating overlapping contributions to oxidative stress and cellular injury. Proline content showed weak and inconsistent correlation with other indicators, suggesting it reflects more genotype-specific osmotic responses rather than a universal thermotolerance marker. Chlorophyll content declined across cultivars, but its correlation with visual damage remained weak (r = 0.114), likely due to asynchronous degradation or regreening responses. Collectively, these findings demonstrate that a multi-index evaluation—integrating visual scoring with physiological parameters—is more robust than relying on single traits. The combination of electrical, malondialdehyde, proline, and chlorophyll measurements provides a comprehensive physiological profile, aiding the identification of heat-tolerant broccoli cultivars and offering practical value for breeding programs aimed at mitigating climate induced stress.

## 4. Discussion

High temperature stress can induce a cascade of physiological disturbances in plants, including oxidative stress, membrane lipid peroxidation, and impaired photosynthetic capacity. Malondialdehyde and electrical conductivity are two widely accepted diagnostic indicators that reflect membrane damage and cellular leakage under stress conditions [21,25]. By quantifying the changes in both electrical conductivity and malondialdehyde content, one can gauge the level of biomembrane damage. In our study, most broccoli varieties exhibited a significant increase in malondialdehyde and electrical conductivity levels after exposure to 40 °C. However, the magnitude of these changes varied markedly between heat-tolerant and heat-sensitive genotypes. For example, the cultivars “Yanxiu” and “Meiqing” maintained relatively low malondialdehyde accumulation and only modest increases in electrical conductivity, suggesting greater membrane stability. In contrast, “Qianghan” and “Zhongqing 16” displayed sharp increases in both malondialdehyde and electrical conductivity, indicating extensive cellular leakage and severe oxidative stress. These findings align with previous studies, which reported that malondialdehyde levels in heat-sensitive broccoli seedlings can increase by up to 100% following exposure to 38–42 °C, whereas tolerant lines maintain more stable profiles [26]. Similarly, electrical conductivity has been validated in multiple studies as a reliable biomarker of membrane damage under abiotic stress [27].

Proline is a small, highly water-soluble amino acid that is ubiquitously present in animals, plants, microorganisms, and cultured cells. Under adverse environmental conditions, plants typically accumulate proline as a protective response, and this trait is often regarded as a key physiological indicator of stress tolerance. Notably, heat-tolerant cabbage varieties tend to accumulate more proline than their heat-sensitive counterparts. The variability in proline responses among broccoli cultivars in our study suggests a more complex, genotype-specific regulatory mechanism. High-temperature stress can lead to malondialdehyde accumulation and significant impairment of membrane function. In our dataset, “Zhongqing 15” and “Zheqing 80” exhibited strong proline accumulation, consistent with an osmoprotective strategy. However, cultivars such as “Qianghan” and even moderately tolerant “Meiqing” showed a decline in proline content after heat treatment. These divergent patterns indicate that although proline serves as a useful stress biomarker, its predictive value for heat tolerance is limited to specific genetic backgrounds.

Chlorophyll is a key photosynthetic pigment essential for plant growth and plays a critical role in various physiological processes. Chlorophyll content directly influences photosynthetic efficiency and serves as a crucial indicator of crop nutritional status, developmental stage, and stress response. In our study, most heat-sensitive cultivars, such as “Zhongqing 16” and “Qianghan”, exhibited a marked reduction in chlorophyll content, accompanied by visible leaf yellowing. In contrast, “Yanxiu” and “Zhongqing 319” retained higher chlorophyll levels, showing only mild senescence and rapid recovery after heat stress. Furthermore, “Yanxiu” and “Meiqing” maintained robust plant structures with minimal leaf damage, stable chlorophyll levels, and no obvious signs of yellowing or senescence. Phenotypic evaluations and survival rate analyses across developmental stages revealed that heat-tolerant lines could quickly resume normal growth after stress relief [28].

At the macro level, correlation analysis confirmed that electrical conductivity had the strongest inverse relationship with phenotypic heat tolerance (r = −0.542). Malondialdehyde was moderately correlated with electrical conductivity (r = −0.440), further supporting its role as a marker of membrane damage. Although proline and chlorophyll are biologically relevant, their correlations with heat tolerance were weaker and inconsistent across genotypes. Our study establishes a cultivar-level physiological benchmark for broccoli seedling-stage heat tolerance, bridging the gap between field-based observations and controlled environment physiological profiling. By integrating visual phenotypic scoring with electrical conductivity, malondialdehyde, chlorophyll, and proline measurements, we propose a scalable and reproducible framework for identifying heat-resilient *Brassica oleracea* germplasm—valuable for breeding programs in the era of climate change.

Previous breeding records and field observations have provided some indications of the relative heat tolerance of certain broccoli cultivars tested in this study. For example, ‘Yanxiu’ and ‘Meiqing’ have been reported by local breeders to exhibit stable performance and minimal yield loss during summer cultivation in northern China, while ‘Qianghan’ and ‘Zhongqing 16’ were often considered less suitable for high-temperature seasons. Our controlled environment results were broadly consistent with these prior assessments: the cultivars previously described as heat tolerant also showed lower malondialdehyde accumulation, more stable chlorophyll content, and higher phenotypic scores under experimental heat stress. Conversely, varieties known to be sensitive in field conditions displayed greater membrane damage and pigment loss in this study. This alignment between historical field observations and controlled environment phenotyping not only validates the reliability of our evaluation system but also suggests its applicability for screening germplasm where field data are scarce or unavailable.

In summary, at 40 °C different crops display a spectrum of damage and stress responses, particularly in terms of electrical conductivity, proline accumulation, malondialdehyde content, and chlorophyll levels. This methodology overcomes the drawbacks of prolonged identification periods, and the inconsistencies and low reproducibility often encountered in field experiments. It offers a robust scientific framework for pinpointing heat resistance and supports the selection and breeding of novel cultivars within the cruciferous vegetable family, including broccoli, kale, and cauliflower.

## 5. Conclusions

This study has developed a system for identifying the heat tolerance of different broccoli varieties. Trait-based Visual Scoring System: A multi-dimensional and structured phenotypic scoring approach (Table 1) has been developed. This method is founded on seven distinct damage traits, enabling a quantitative comparison of the responses of different varieties during the seedling stage. Integrated Evaluation Framework: By integrating physiological indicators with structured phenotypic grading and correlation analysis, we have constructed a more comprehensive and reproducible heat tolerance screening system. The observed negative correlation between electrical conductivity and heat tolerance can be regarded as a valuable criterion for variety selection. This technique addresses the challenges associated with time consuming, costly, and unreliable field-based identification procedures. Cultivar-specific Profiling: We have systematically evaluated 14 broccoli cultivars currently in use in China, identifying the key physiological patterns and damage types associated with heat tolerance and sensitivity. These findings hold practical implications for breeding programs.

## Figures and Tables

**Table 1:** The information of 14 broccoli cultivars planted in China since 2014.

Accessions	Varieties	Generations	Origins	Maturity
B1	Naihanyouxiu	F1 Hybrid	SAKATA, Japan	Mid–early maturity
B2	Yanxiu	F1 Hybrid	SAKATA, Japan	Mid–late maturity
B3	Qianghan	F1 Hybrid	SEMINIS, America	Mid–late maturity
B4	Feicui 5	F1 Hybrid	SYNGENTA, China	Middle maturity
B5	Lvxiong 90	F1 Hybrid	TOKITA, Japan	Late maturity
B6	Guowang11	F1 Hybrid	ZHAOFENG, China	Surplus late maturity
B7	Zheqing80	F1 Hybrid	MITSUO, China	Surplus late maturity
B8	Zhongqing11	F1 Hybrid	IVF-CAAS, China	Surplus early maturity
B9	Zhongqing 15	F1 Hybrid	IVF-CAAS, China	Mid–late maturity
B10	Zhongqing 16	F1 Hybrid	IVF-CAAS, China	Early maturity
B11	Zhongqing 318	F1 Hybrid	IVF-CAAS, China	Surplus late maturity
B12	Zhongqing 319	F1 Hybrid	IVF-CAAS, China	Late maturity
B13	Meiqing	F1 Hybrid	MITSUO, China	Mid–late maturity
B14	Meiao7172	F1 Hybrid	MITSUO, China	Mid–late maturity

**Table 2:** Summary of diurnal heat stress regimes used in previous studies on Brassicaceae crops.

Species	Day/Night Temperature (°C)	Light Duration	Night Duration (h)	Study
Broccoli	40/36	16	8	[17]
Oilseed rape (*Brassica napus* L.)	36/20	16	8	[8]
Arabidopsis thaliana	38/28	12	12	[18]
Chinese Cabbage (*Brassica rapa* L. ssp. *pekinensis*)	45/35	16	8	[19]
Broccoli	40/36	16	8	This study

**Table 3:** Trait-based phenotypic damage scores for Broccoli cultivars under heat stress.

Accessions	Varieties	Wilting	Drying	Regreening	Collapse	Green Loss	Heart Leaf Change	Apical Damage	Final Score
B1	Naihanyouxiu	3	3	2	2	3	3	2	2.57
B2	Yanxiu	3	2	2	3	2	3	2	2.43
B3	Qianghan	4	4	4	4	4	5	4	4.14
B4	Feicui 5	3	3	3	3	3	4	3	3.14
B5	Lvxiong 90	4	4	4	4	4	5	4	4.14
B6	Guowang 11	3	3	3	3	3	4	3	3.14
B7	Zheqing 80	3	3	3	3	3	4	2	3.00
B8	Zhongqing 11	4	4	5	5	5	5	5	4.71
B9	Zhongqing 15	3	3	3	3	3	4	3	3.14
B10	Zhongqing 16	5	4	5	5	5	5	5	4.86
B11	Zhongqing 318	3	3	3	3	3	4	3	3.14
B12	Zhongqing 319	3	3	3	3	3	4	3	3.14
B13	Meiqing	3	3	2	3	2	3	2	2.57
B14	Meiao 7172	3	3	3	3	3	3	3	3.00

## Data Availability

The data presented in this study are available on request from the corresponding author.
